# Genetic determinants of syndactyly: perspectives on pathogenesis and diagnosis

**DOI:** 10.1186/s13023-022-02339-0

**Published:** 2022-05-12

**Authors:** Afraah Cassim, Dineshani Hettiarachchi, Vajira H. W. Dissanayake

**Affiliations:** Human Genetics Unit, Faculty of Medicine, University of Colombo, 25, Kynsey Road, Colombo, Sri Lanka

**Keywords:** Syndactyly, Limb, WNT-BMP-FGF, Synostosis, Syndactyly classification, Genetic screening

## Abstract

The formation of the digits is a tightly regulated process. During embryogenesis, disturbance of genetic pathways in limb development could result in syndactyly; a common congenital malformation consisting of webbing in adjacent digits. Currently, there is a paucity of knowledge regarding the exact developmental mechanism leading to this condition. The best studied canonical interactions of Wingless‐type–Bone Morphogenic Protein–Fibroblast Growth Factor (WNT–BMP–FGF8), plays a role in the interdigital cell death (ICD) which is thought to be repressed in human syndactyly. Animal studies have displayed other pathways such as the Notch signaling, metalloprotease and non-canonical WNT-Planar cell polarity (PCP), to also contribute to failure of ICD, although less prominence has been given. The current diagnosis is based on a clinical evaluation followed by radiography when indicated, and surgical release of digits at 6 months of age is recommended. This review discusses the interactions repressing ICD in syndactyly, and characterizes genes associated with non-syndromic and selected syndromes involving syndactyly, according to the best studied canonical WNT-BMP-FGF interactions in humans. Additionally, the controversies regarding the current syndactyly classification and the effect of non-coding elements are evaluated, which to our knowledge has not been previously highlighted. The aim of the review is to better understand the developmental process leading to this condition.

## Background

Syndactyly, devised from the Greek word ‘syn’ meaning ‘together,’ and ‘dactyly’ meaning ‘digits’, is a congenital malformation, where there is failure of separation (aberrant webbing) between adjacent digits during embryological limb formation [1, 2]. It is one of the most common hereditary deformities, occurring in 1 in 2000 live births, and has twice the occurrence in males than in females. There is seen to be half the proportion of cases to have bilateral syndactyly, whilst 57% of cases involve the third web space [3]. The underlying aetiology for syndactyly is mostly genetic but causes such as maternal smoking, lower nutritional and economic status have also been elucidated [3].

This phenotype is present as soft tissue and/or bony fusions, or further characterized as complete fusions if adjacent digits are fused until the fingertips, else, as incomplete. It can be present in isolation or as a feature of more than 300 recognized syndromes. Non-syndromic syndactyly is currently classified into 9 main types and subtypes, some subtypes possessing loci with genes to be identified [1, 4, 5]. Syndactyly may also be a feature in syndromes including Acrocephalosyndactyly, Fraser or even associated with other digit abnormalities (Ex: polydactyly). A conglomeration of pathways for syndactyly pathogenesis have been stipulated, involving a plethora of genes [6–10].

The majority of them are diagnosed clinically, followed by radiographs when indicated to help determine if syndactyly is complete/complex [11]. Surgical release of joined digits is the current therapy, albeit recommended earlier release at 6 months to prevent angulatory rotational distortions of some digits. Prenatal diagnosis of syndactyly is difficult and hand syndactyly can be mistaken for clenched fists [12].

Through this article, we attempt to better understand the developmental process underlying this condition and the genes involved.

## Digit separation interactions in normal vs. syndactyly phenotypes

Human limb structure regulation is controlled by two signaling centers; the zone of polarizing activity (ZPA controlling overall patterning in the anterior–posterior axis) and the apical ectodermal ridge (AER for limb growth) [13]. Different interactions including WNTs, Bone Morphogenetic Proteins (BMPs), Fibroblast Growth Factors (FGFs), and Retinoic acid metabolites (RA) have been elucidated to have a role in the ICD in the nascent autopod. Initial autopod formation is stipulated as the interactions arising from the FGF and WNT signaling.

*The canonical WNT signaling* in particular has been studied extensively. This is also controlled by the balance of Sonic hedgehog in the ZPA regions. Bone morphogenetic protein 2 (BMP2), 4, and 7 are expressed in the interdigit and they are associated with apoptosis in the interdigits [14, 15]. The BMPs produce retinoic acid metabolites, which may further downregulate FGF signaling. It is known that FGF signaling is a potential survival pathway with anti-apoptotic signals. They are represented mainly by *FGF4* and *FGF8* in the AER promoting growth of the underlying mesenchyme [16]. Retinoic acid, may induce ICD directly distally, but is thought to be induced by the BMP-FGF axis indirectly to induce ICD proximally [2, 17]. Together, the signaling interactions of WNT-BMP-FGF and retinoic acid are vital for autopod digit separation. To date, there is no specified axis of syndactyly pathogenesis. However, the absence of ICD during the 7th to 8th weeks of gestation in the interdigital mesenchyme involving the WNT-BMP-FGF axis seems to be a highly conjectured pathway for the pathogenesis of soft tissue syndactyly [17–19]. The first steps in cutaneous syndactyly are described as the overexpression of the WNT canonical pathway, or the suppression of BMPs in the interdigital mesenchyme. Lower levels of BMPs, which normally positively regulate interdigital apoptosis, now leads to an overexpression of FGF8 causing increased anti-apoptotic signals in the AER. This can indirectly inhibit retinoic acid and finally repress its function in the extracellular matrix (ECM), causing perturbed ICD inducing syndactyly phenotypes [7, 13, 17, 19].

*Non canonical WNT-PCP pathway* While FGF8 modulates only rapid disorganized movement of cells, WNT5A (3p14.3) expressed in the AER induces polarized orientation of cells via the WNT-PCP pathway and this was observed in cell culture and is known to be required for ICD [20, 21]. Most focus for the underlying pathology of complex syndactyly, is the WNT-BMP-FGF8 pathway. It is reported that ICD occurs at E12.5-E13.5 in mouse cell lines [22]. In a study by Zhu et al., WNTless; a key regulator of WNT trafficking was tested. The WNTless mutants (*WI*^*Shh-Cre*^) show webbing of cartilage before E12.5, suggesting that abrogated ICD does not account for cartilage webbing in WNTless mutant mice. This deficiency of *WNT5a* could outride the Shh regulation (occurs half a day later), and could be a better suited cause for digit malformations in the WNTless mutant mice [22]. *HOXD13* mutants displayed downregulated WNT5a signaling, which could mean WNT5a gradient in chondrocyte cell polarity could be an imperative aetiology [10, 22]. Hence a combined effect of both FGF and non-canonical WNT-PCP pathways is postulated for normal cell orienting, especially for synostosis syndactyly commonly seen in Malik–Percin type or complex syndactyly [20, 23].

*Notch Signaling* There is control of ICD by modulating the level of FGF8 expressing cells in the AER. This was exemplified in murine models deficient in the Notch ligands, Serrate or Jagged2, where syndactyly was observed as a result of expanded FGF8 domain and substantial decrease of BMPs in the interdigital tissue [6, 13].

*A Disintegrin And Metalloproteinase with Thrombospondin Motifs (ADAMTS) Metalloproteases* Proteolytic activity of the proteases ADAMTS5 (21q21.3), ADAMTS9 (3p14.1) and ADAMTS20 (12q12) have been postulated to be vital for completion of ICD. As ADAMTS 5/20 does not act upstream, this leaves the BMP/FGF axis unaffected. Thus, the ADAMTS Metalloproteases with cleaved Versican, prove as a promising alternative independent pathway to the BMP/FGF axis, and act concurrently to produce ICD in the developing limb bud [2, 8]. Finally, Hedgehog signaling critically influences the distribution of other signaling pathways. Interactions in Notch signaling and the ADAMTS pathway also seem to contribute to soft tissue syndactyly in humans but are less prioritized [8, 24]. This might be since the deletion of Notch1 is lethal before limb bud stages, its role in development remains elusive. Additionally, only animal models have been used to observe syndactyly pathogenesis in Notch and ADAMTS. Collectively, the perturbation of ICD may account for simple (cutaneous) syndactyly. However, it is questionable that complex (bony) syndactyly phenotypes arise solely from perturbed ICD, prompting further investigation [2].

The importance of analyzing genes in pathogenesis will help in understanding the genes involved in autopod formation, as well promote analyzing other gene–gene and protein–protein interactions which may contribute to syndactyly. To date, the canonical WNT pathway only justifies pathogenesis of cutaneous syndactyly in the developing limb bud, and does not account fully for synostosis syndactyly; such as the Malik Percin type. Since interdigital webbing occurs at day E12.5 before ICD in mice models, the WNT-PCP non-canonical pathway seems to be a promising mechanism for aberrant mesenchymal cell migration in synostosis syndactyly phenotypes [10, 22].

## Syndromic syndactyly genetic determinants

The complexity of syndactyly classification is augmented since it can exist in 300 or more syndromic forms [1, 3]. Selected syndromic phenotypes having postulated mechanisms for pathogenesis, are described below and listed in Table 1.

**Table 1:** Genetic determinants for selected syndromic syndactyly phenotypes

Gene	Type of syndrome	OMIM	Common variant/s	Inheritance	Clinical presentation	Postulated contribution to pathogenesis	Key references
**1 (a) Genetic determinants in coding regions**							
*ROR2*	Brachydactyly type B (BDB1)	113000	c.1324C.T; p.R441X	AD	Brachydactyly characterized by hypoplasia/aplasia of distal phalanges in combination with distal symphalangism, fusion of carpal/tarsal bones, and partial cutaneous syndactyly	Facilitate WNT Overexpression	[20, 40]
*SOST*	Sclerosteosis	269500	Nonsense mutations	AR	Presence of asymmetric cutaneous syndactyly of the index and middle fingers in many cases. The jaw has an unusually square appearance		[29, 41]
*LRP4*	Sclerosteosis, 2	614305	c.3508C > T; p.R1170W and c.3557G > C; p.W1186S	AD, AR	Progressive skeletal overgrowth. Syndactyly is a variable manifestation		[26, 28, 42]
*GLI3*	Pallister–Hall syndrome	146510	Haploinsufficiency, c.1468_1469insG and c.1007_1008dupAC	AD	Hypothalamic hamartoma, pituitary dysfunction, central polydactyly, and variable degree of syndactyly	BMP suppression	[30, 43, 44]
	Greig cephalopolysyndactyly syndrome	175700	c.2374C > T; p.Arg792*	AD	Frontal bossing, scaphocephaly, and hypertelorism associated with pre- and postaxial polydactyly and variable syndactyly		
*LMBR1*	Triphalangeal thumb-polysyndactyly syndrome	174500	Position 287 on ZRS enhancer	AD	Thumb in this malformation is usually opposable and possesses a normal metacarpal. Variable degree of syndactyly		[31, 32, 45]
*DHCR7*	Smith–Lemli–Opitz syndrome	270400	c.453G > A; p.W151X	AR	Affects multiple body systems with Syndactyly of toes 2 and 3 being a common finding		[33, 46]
*RAB23*	Carpenter syndrome	201000	Homozygous nonsense/frameshift pathogenic variants c.434 T > A; p.L145X	AR	Craniosynostosis, polysyndactyly, obesity, and cardiac defects		[39, 47–49]
*FGFR2*	Apert syndrome	101200	c.755C > G; p.S252W or c.758C > G; p.P253W	AD	Craniosynostosis, midface hypoplasia, and syndactyly of the hands and feet with a tendency to fusion of bony structures	FGF Overexpression	[7, 34, 50–52]
*FGFR1/FGFR2*	Pfeiffer syndrome	101600	*FGFR1* – p.P252R	AD	Type 1, the classic syndrome, is compatible with life and consists of craniosynostosis, midface deficiency, broad thumbs, broad great toes, brachydactyly, and variable syndactyly		
*TWIST1;FGFR3*	Saethre–Chotzen syndrome	101400	*FGFR3* p.P250R, deletion of *TWIST1*	AD	Craniosynostosis, facial dysmorphism, and hand and foot abnormalities. The degree of syndactyly is also variable		[35, 36, 53]
*HOXD13*	Brachydactyly-syndactyly syndrome	610713	Polyalanine constriction, c.950A > G; p.Q317K	n.r	Brachydactyly and syndactyly (partial cutaneous webbing) in association with oligodactyly	Retinoic acid suppression	[10, 37]
*LRP4*	Sclerosteosis, 2	614305	c.1151A > G; p.Tyr384Cys	AD, AR	Progressive skeletal overgrowth. Syndactyly is a variable manifestation	Repression of Notch signaling	[54]

Other loci/variants	Clinical presentation	Postulated contribution to pathogenesis	Key references
**1 (b) Genetic determinants in non-coding regions**			
Intron *EMID2*	Holoprosencephaly spectrum disorder and severe upper limb syndactyly	Ectopic SHH expression	[55]
Intron 5 *LMBR1*	Cutaneous syndactyly without polydactyly	Decreased ICD	[45]
Intron *IRF6*	Van der Woude syndrome (MIM: 119,300)	Unclassified	[56]
Exonization of 22 intronic *YY1AP1*	Grange syndrome (MIM: 602,531) with complete cutaneous syndactyly. Fingers: third, fourth, and fifth finger and third/fourth finger of the right hand. Toes: bilateral cutaneous syndactyly of her second/third toes	Unclassified	[57]
Intron 6 *KATNB1*	Congenital microcephaly, lissencephaly, short stature, polysyndactyly, and dental abnormalities	Unclassified	[58]
Intron 8 *FGFR2*	Apert syndrome (MIM 101,200)	Unclassified	[59]
IVS8-1G > C variant *DHCR7*	Smith-Lemli-Opitz syndrome (MIM 270,400)	BMP suppression	[60]
Pre-ZRS region	TPS (MIM: 174,500)	Ectopic expression	[61]

*AR* autosomal recessive, *AD* autosomal dominant, *XLR* X-Linked recessive, *n.r* not reported due to insufficient evidence, *EMID2* EMI domain containing 2, *LMBR1* Limb development membrane protein 1, *IRF6* Interferon regulatory factor 6, *YY1AP1* YY1 associated protein 1, *KATNB1* Katanin,p80 subunit B1, *FGFR2* Fibroblast growth factor receptor 2, *TPS* Thumb polysyndactyly syndrome

### Variants in coding regions

Some of the syndromes are mentioned below with their documented involvement in each interaction for canonical pathogenesis, but one must note that many other syndromes are also present. The genetic variants for each syndrome are listed where found in Table 1*.*

Some of the main WNT overexpressing syndromes observed include Brachydactyly type B (BDB1) (MIM 113000), Sclerosteosis (MIM 269500) and Sclerosteosis, 2 (MIM 614305).

BDB1 is mapped to the *ROR2* gene, (9q22.31) encoding a co-receptor to WNT ligands, such as WNT5a. It is postulated that ROR2 may implicate canonical WNT signaling since a striking resemblance of the CRD of ROR2 and the WNT binding domain of Frizzled receptors has been identified [25]. It is also seen to be involved in the non-canonical WNT-PCP interactions, since mice with mutant ROR2 produce similar phenotypes in comparison to WNT5a mutant mice [20]. Sclerosteosis 2 has been mapped to the *LRP4* gene where there is direct interaction between sclerostin and LRP4, and LRP4 facilitates sclerostin mediated WNT inhibition; which is implicated in *LRP4* mutants [26, 27]. Additionally, LRP4 interactions with the Notch pathway is known to b evolutionarily conserved and a homozygous missense variant in the highly conserved EGF-2 calcium binding domain of the *LRP4* gene was also hypothesized to have abrogated Notch signaling [28].

Finally, in Sclerostosis, loss of function *SOST* gene variants may enable sclerostin to inhibit the LRP5/6 and frizzled receptor complex, thereby leading to phosphorylation and downregulation of β-catenin resulting in the inhibition of WNT signaling interactions [29].

The main syndromes seen to promote BMP suppression include Pallister-Hall syndrome (PHS) (MIM 146510) and Greig cephalopolysyndactyly syndrome (MIM 175700), Triphalangeal thumb-polysyndactyly syndrome (TPS) (MIM 174500), Smith-Lemli-Opitz syndrome (SLOS) (MIM 270400) and Carpenter Syndrome (MIM 201000). PHS and GCPS are both mapped to the *GLI3* gene. However, Pallister-Hall syndrome is associated with central polydactyly while GCPS involves pre and post axial polydactyly. The *GLI3* gene (7p14), encodes a transcription factor that is a bifunctional downstream modulator of SHH signaling; which is implicated in both syndromes. Most *GLI3* gene variants are associated with haploinsufficiency of the *GLI3* gene, which eventually cause the skewing of GLI3 Activator (GLIA) or Repressor (GLIR) formation [30]. Common genetic determinant for TPS includes duplications spanning the *LMBR1* gene [7, 31, 32], and the *RAB23* gene (6p12.1-q12), encodes a negative regulator of the signaling in sonic hedgehog (SHH), perturbed in Carpenters syndrome. As for SLOS, the gene encoding the enzyme 7-dehydrocholesterol (7-DHC) reductase (*DHCR7*: 11q13.4), is known to be deregulated. The *DHCR7* gene is hypothesized to orchestrate cholesterol moiety and SHH signaling, which governs the severity of syndactyly phenotypes [33].

Syndromic dactylies shown to promote FGF8 overexpression mainly include Apert Syndrome (MIM 101200), Pfeiffer Syndrome (MIM 101600) and Saethre-Chotzen syndrome (MIM 101400) syndrome. The FGFR2 gene variants implicates the function of this receptor in both Apert and Pfeiffer syndromes. The P253W mutant may perturb ligand-binding specificity to FGF10 resulting in overstimulation of FGF8 via the FGF10-FGF8 loop in the ectoderm [7, 34]. As for Saethre-Chotzen syndrome, *TWIST1* and *FGFR1/3* genes have been mapped to this syndrome. *TWIST1* is classified as an upstream regulator of FGFs, thus modulating FGF expression [35, 36].

Finally, the Brachydactyly-syndactyly syndrome (MIM 610713) associated with variants in the *HOXD13* gene (polyalanine constrictions, and missense variants) [10, 37]. It is postulated that *HOXD13* mutants*,* suppresses retinoic acid (RA) in the ECM.

### Variants in non coding regions

The importance to screen the gene and its flanking regions remains imperative, since alternative splice variants in non-coding regions may lead to exon skipping, and epigenetic influences which underlie protein malformation [1, 5]. The presence of modifier genes has been postulated to influence phenotypes in gene variants of *p63* [38] and *RAB23* [39]*,* which may underlie causes for intrafamilial variability. A number of intronic loci (Table 1), have been identified in syndactyly phenotypes, although most require classification of their pathway.

## Non-syndromic syndactyly genetic determinants

Together, 9 types of non-syndromic syndactyly (I-IX), according to their phenotypical diversity have been classified (Table 2), with the respective gene/loci [1]. Some of these identified genes include *HOXD13, FBLN1, GJA1, LMBR1, LRP4, GREM1* (BMP antagonist)*, FGF16* and recently discovered *BHLHA9* [1, 62–65].

**Table 2:** Genes/loci with postulated pathogenesis for non-syndromic syndactyly

Type of non syndromic syndactyly		Locus/gene	OMIM	Common variant/s	Inheritance	Clinical presentation	Postulated contribution to pathogenesis	Key references
I-a	ZD1; Zygodactyly; Weidenreich type	3p21.31	609815	n.r	AD	Bilateral, symmetrical, Fingers: Normal Toes: 2/3 only	n.r	[64]
I-b	SD1; Lueken type	2q34-q36	185900	n.r	AD	Usually, bilateral Fingers: 3/4 Fingers, cutaneous/bony Toes: 2/3 Toes, cutaneous	n.r	[68, 69]
I-c	Montagu type	2q31-q32 *HOXD13*	n.r	c.917G > A; p.R306Q, c.916C > G; p.R306G	AD	Typically, bilateral Fingers: 3/4 Fingers only, cutaneous/bony Toes: Normal	Suppression of Retinoic Acid	[70]
I-d	Castilla type	n.r	n.r	n.r	AD	Bilateral Fingers: Normal Toes: 4/5 Toes only, cutaneous	n.r	[71]
II-a	SPD1; Vordingborg type	2q31; *HOXD13*	186000	Polyalanine repeat expansions, frameshift deletions, 2q31.1 microdeletion and G11A missense	AD	Fingers: SPD, mesoaxial (3/4 fingers) Toes: SPD, postaxial (4/5 toes)	Suppression of Retinoic Acid	[72]
II-b	SPD2; Debeer type	22q13.3; *FBLN1*	608180	t(12;22) (p11.2;q13.3)	AD	Fingers: SPD is central and postaxial Toes: Postaxial syndactyly	Increased expression of FGFR8	[73]
II-c	SPD3; Malik type	14q11.2-q13	610,234	n.r	AD	Fingers: SPD is central Toes: SPD postaxial	n.r	[65]
III	SDTY3; Johnston-Kirby type	6q21-q23; *GJA1*	186100	nt427G > A and c.T274C; p.Y92H	AD	Fingers: Complete, bilateral syndactyly of 4/5 Fingers; fifth finger short, middle digit missing or underdeveloped Toes: Normal	Reduces downstream BMP2 expression, contributing to overexpression of the FGF4 and FGF8	[74–76]
IV-a	SDTY4; Haas type	7q36; ZRS (*LMBR1)*	186200	Heterozygous variants in the SHH regulatory element (ZRS), and duplications	AD	Complete and Bilateral, often polydactyly associated Fingers: All fingers webbed; pre-/post-axial polydactyly, cup-shaped hand Toes: Normal	Alter the ZRS control/limb-specific SHH expression	[77, 78]
IV-b	Andersen-Hansen type	n.r	n.r	n.r	n.r	Fingers: All fingers webbed; pre-/post-axial polydactyly, cup-shaped hand Toes: Variable webbing of toes with polydactyly	n.r	[79]
V	SDTY5; Dowd type	2q31; *HOXD13*	186300	c.950A > G; p.Q317R, and polyalanine expansions	AD	Complete Fingers: 4/5 Fingers with metacarpals fusion; hypoplastic metacarpals 4/5 Toes: Mesoaxial webbing	Suppression of Retinoic Acid	[37, 80]
VI	Mitten type	n.r	n.r	n.r	AD	Unilateral Fingers: 2/5 Fingers Toes: 2/5 Toes	n.r	[81]
VII-a	Cenani-Lenz type; spoon-hand type	11p12–p11.2; *LRP4*	212780	Missense variations are most common, c.1117C > T; p.R373W	AR	Fingers: Total synostotic syndactyly with metacarpals fusion, spoon-head shape Toes: Total synostotic syndactyly with metatarsals fusion	WNT overexpression /Postulated to cause repression of Notch signaling	[82]
VII-b	Oligodactyly type	15q13.3; *GREM1-FMN1*	n.r	1.7 Mb duplication spanning both the *GREM1* and *FMN1* 1 genes	AD	Fingers: Few deformed digits Toes: Variable syndactyly of toes	BMP antagonist GREM1(usually repressed by FGF); is active in mutant forms, suppressing BMP activity	[83]
VIII-a	Orel-Holmes type	Xq21.1; *FGF16*	309630	Nonsense variants p.R179X and p.S157X in exon 3	X-R	Fingers: 4/5 Metacarpal fusion Toes: Normal	Impair FGF16-FGFR1 interactions	[84, 85]
VIII-b	Lerch type	n.r	n.r	n.r	AD	Fingers: 4/5 Metacarpal fusion Toes: Normal	n.r	[1]
IX	MSSD; Malik-Percin type	17p13.3/ BHLHA9	609432	c.311T > C; p.Ile104Thr	AR	Fingers: Mesoaxial synostotic syndactyly with phalangeal reduction Toes: Preaxial webbing; distal phalangeal hypoplasia	Ectopic FGF8 expression restrict BMP suppression	[63, 86, 87]

*AR* autosomal recessive, *AD* autosomal dominant, *XLR* X-Linked recessive, *n.r* not reported due to insufficient evidence, *SPD* synpolydactyly

However, some controversies regarding non-syndromic syndactyly classification have been identified. A report by Qattan et al. identified a case with cutaneous syndactyly occurring in digits two/five or three/five without polydactyly. This finding of isolated syndactyly without polydactyly is similar to type III syndactyly, caused by variants in the *GJA1* gene and has been observed in digits four/five or three/five. However, although similar to type III, the authors observed a variant to be present in the ch7q36.3 (*LMBR1* region) which is syntenic to the Hammertoe locus in mice of the similar phenotype [45]. This may highlight a case of genetic heterogeneity, and supports the notion that this type may be polygenic.

In another two reports regarding type IX syndactyly which is characterized by osseous synostosis of the third and fourth metacarpals, whereby slightly different phenotypes have been associated. Weinrich et al. highlighted a case of MSSD involving the second and third proximal phalanges which is different from Malik’s original classification and attributed it to being a novel non-syndromic type [66]. However, this notion was dismissed and re-classified by Malik as being a type of non-syndromic type IX associated with the *BHLHA9* gene [63]. The second report, presented a case of MSSD with a triangular proximal phalanx of the second and third metacarpal similar to Weinrich’s case report, in addition with ulnar hemimelia (shortening), a novel finding [67]. Genetic testing was not performed in both MSSD cases, and conclusions on classification were based on hand radiography. If it maps to the same gene, this could be a form of phenotypic heterogeneity associated with the *BHLHA9* gene. Hence it remains imperative to genetic testing to be able to further evaluate these inconsistencies.

## Application to genetic screening

**Fig. 1:**
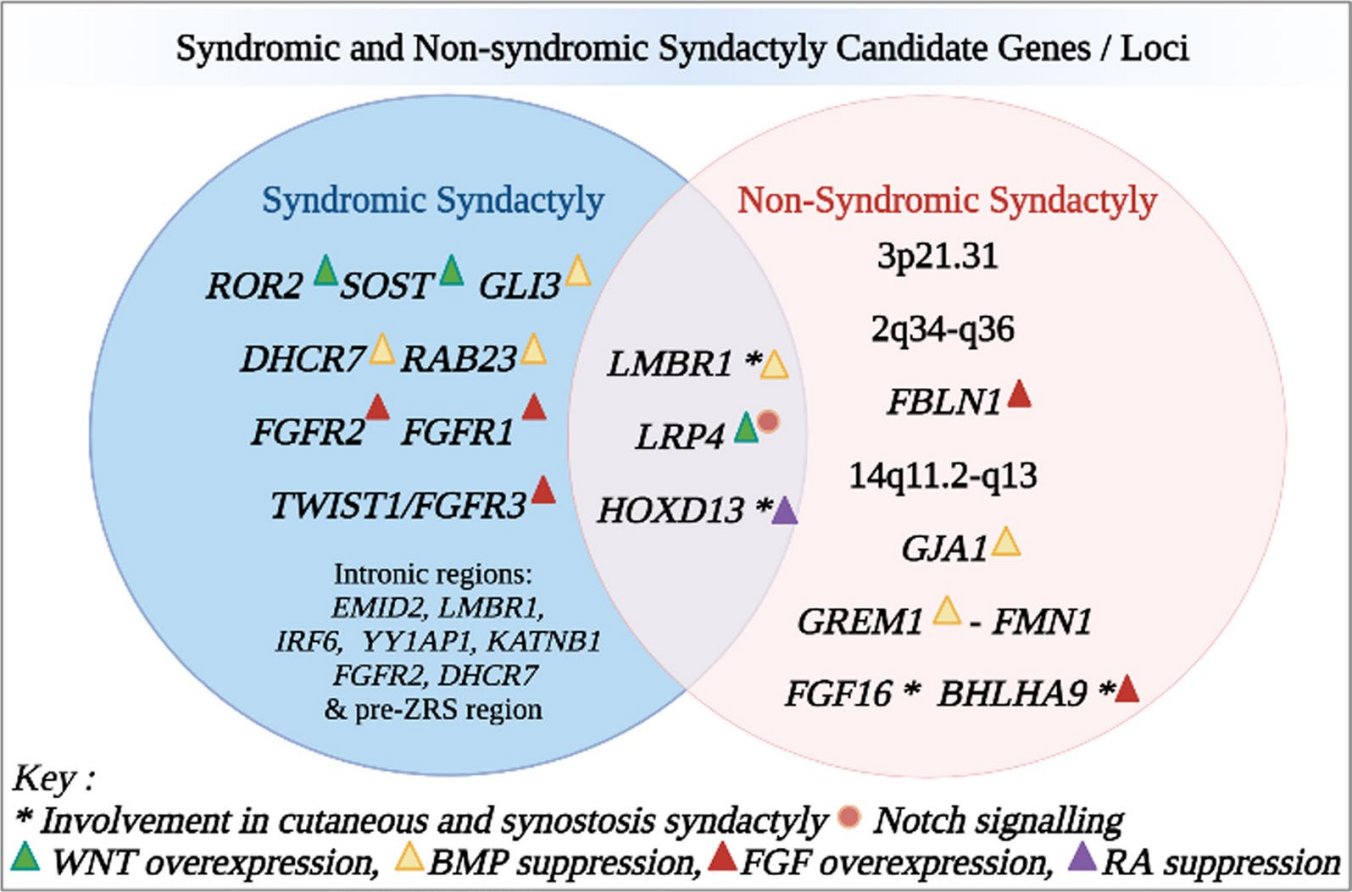
Representation of genes and loci in syndromic and non syndromic syndactyly analyzed in this review. These variants are further characterized according to involvement in documented syndactyly pathogenesis interactions. *WNT* Wingless‐type integration site family, *BMP* Bone Morphogenic Protein, *FGF* Fibroblast Growth Factor, *RA* Retinoic Acid

Emerging evidence into genes in syndactyly may provide an alternative gene screening panel as a diagnostic; especially prenatally when radiographic diagnosis is almost impossible. The *GJA1, HOXD13* and *FGF16* genes involved in non-syndromic syndactyly, involve the fourth webspace and can offer diagnostic choice in the pretext of prenatal screening; to prevent angulatory deformities later [3]. The *LMBR1, LRP4* and *HOX13* genes are seen to be deregulated in both syndromic and non-syndromic forms (Fig. 1). Additionally, *HOXD13* may be able to modulate both cutaneous and osseous syndactyly pathways. Hence, inclusion of these genes in gene panels will expand diagnosis criteria. During diagnosis, if no conclusion is reached on gene testing, it can be important to screen flanking regions, and intronic regions. Not to forget the presence of syndactyly itself, in syndromes, can sometimes be a conclusive factor to categorize the syndrome (Ex: syndactyly of the second/third toes in SLOS) [46].

## Further characterization of genetic determinants

Non syndromic syndactyly is thought to be well characterized, however there has been controversy over the associated MSSD phenotype, where both case reports did not include genetic testing for the *BHLHA9*, so the possibility of phenotypic heterogeneity cannot be excluded [63, 67]. Genetic Heterogeneity; although Cenani Lenz phenotype has been attributed to the gene *LRP4*, there have been reports with the same phenotype in *APC* gene [18]. Additionally, syndactyly Type III in Qattan’s study may prove another locus in addition to *GLI3* [45]. Furthermore, since BMPs are secreted factors, gene expression patterns will not fully correlate with functional perspectives. Hence, protein distribution also needs to be considered. Moreover, understanding gene–gene interactions, non-coding variants and epigenetics further prompts better characterization of syndactyly phenotypes.

For syndactyly of the first and fourth webspaces, earlier release at approximately 6 months of age is advised [88]. Hence genes associated with non-syndromic Castilla type and Lerch type can be prioritized for further study, in addition to other known genes in syndromic syndactyly involving these webspaces. The complexity of syndactyly is furthered as one gene may involve more than one pathway. The *LRP4* gene variants are known to be involved in overexpression of WNT, but also variants showing abrogated Notch signaling [28]. On the other hand, majority of syndromic syndactyly phenotypes remain unclassified in terms of likely involvement in pathways [73, 89–92].

Therapeutic targets from Mendelian traits and genome-wide association studies (GWASs) are more likely to be successful in clinical studies for multifactorial diseases [93]. Thus, efforts to analyze genes in this regard seem promising.

## Conclusion

Autopod development is a tightly regulated process involving a plethora of genes. A classical belief is that loss of interdigital cell death by the WNT canonical pathway causes syndactyly. However, many genes with unclassified pathways still exist, with most other pathways being investigated in only animal studies. We propose that a conglomeration of genes in the canonical and non-canonical WNT signaling, Notch signaling, ADAMTS metalloprotease pathways and non-coding regions may work in concert to establish normal development of the limb bud. Candidate gene studies on *HOXD13, LMBR1, FGF16, BHLHA9*, to understand their contribution to both cutaneous and synostosis syndactyly phenotypes, could enhance screening. The genes *GJA1, HOXD13* and *FGF16* along with other genes involving either the first and fourth webspaces may also be prioritized as prenatal genetic testing here is urgently necessary. Collectively, the translation of these findings to human research, especially pathway mapping, could further resolve the genetic underpinnings associated with syndactyly. Furthermore, it will aid in the management of syndactyly and facilitate biomarker discovery.

## Data Availability

Not applicable.

## Abbreviations

ADAMTS: A disintegrin and metalloproteinase with thrombospondin motifs
AER: Apical ectodermal ridge
BMP: Bone morphogenic protein
CLSS: Cenani–Lenz syndactyly syndrome
CRD: Cysteine-rich domain
DHCR7: 7-Dehydrocholesterol (7-DHC) reductase
ECM: Extracellular matrix
FGF: Fibroblast growth factor
GLIA: GLI3 activator
GLIR: GLI3 repressor
GCPS: Greig cephalopolysyndactyly syndrome
IHH: Indian hedgehog
ICD: Interdigital cell death
LRP4: Low-density lipoprotein receptor-related protein 4
MDDS: Mesoaxial synostotic syndactyly syndrome
MS4: Synostosis of the fourth and fifth metacarpals
PCP: Planar cell polarity
RA: Retinoic acid
SCS: Saethre–Chotzen syndrome
SHH: Sonic Hedgehog
SLOS: Smith–Lemli–Opitz syndrome
WNT: Wingless‐type integration site family
ZPA: Zone of polarising activity
ZRS: Zone of polarizing activity regulatory sequence

## References

[1] Malik S (2012). Syndactyly: phenotypes, genetics and current classification. Eur J Hum Genet.

[2] Hernández-Martínez R, Covarrubias L (2011). Interdigital cell death function and regulation: new insights on an old programmed cell death model. Dev Growth Differ.

[3] Jordan D, Hindocha S, Dhital M, Saleh M, Khan W (2012). The epidemiology, genetics and future management of syndactyly. Open Orthop J..

[4] Ahmed H, Akbari H, Emami A, Akbari MR (2017). Genetic overview of syndactyly and polydactyly. Plast Reconstr Surg.

[5] Umair M, Ahmad F, Bilal M, Abbas S (2018). Syndactyly genes and classification: a mini review. J Biochem Clin Genet..

[6] Montero JA, Hurle JM (2010). Sculpturing digit shape by cell death. Apoptosis.

[7] Al-Qattan MM (2019). A review of the genetics and pathogenesis of syndactyly in humans and experimental animals: a 3-step pathway of pathogenesis. Biomed Res Int..

[8] McCulloch DR, Nelson CM, Dixon LJ, Silver DL, Wylie JD, Lindner V (2009). ADAMTS metalloproteases generate active versican fragments that regulate interdigital web regression. Dev Cell.

[9] Bocci F, Onuchic JN, Jolly MK (2020). Understanding the principles of pattern formation driven by Notch signaling by integrating experiments and theoretical models. Front Physiol..

[10] Kuss P, Kraft K, Stumm J, Ibrahim D, Vallecillo-Garcia P, Mundlos S (2014). Regulation of cell polarity in the cartilage growth plate and perichondrium of metacarpal elements by HOXD13 and WNT5A. Dev Biol.

[11] Ermito S, Dinatale A, Carrara S, Cavaliere A, Imbruglia L, Recupero S (2009). Prenatal diagnosis of limb abnormalities: role of fetal ultrasonography. J Prenat Med.

[12] Li Y, Ma D, Sun Y, Meng L, Wang Y, Jiang T (2018). Apert syndrome with FGFR2 758 C > G mutation: a Chinese case report. Front Genet.

[13] Kaltcheva MM, Anderson MJ, Harfe BD, Lewandoski M (2016). BMPs are direct triggers of interdigital programmed cell death. Dev Biol.

[14] Pajni-Underwood S, Wilson CP, Elder C, Mishina Y, Lewandoski M (2007). BMP signals control limb bud interdigital programmed cell death by regulating FGF signaling. Development.

[15] Macias D, Gañan Y, Ros MA, Hurle JM (1996). In vivo inhibition of programmed cell death by local administration of FGF-2 and FGF-4 in the interdigital areas of the embryonic chick leg bud. Anat Embryol.

[16] Boulet AM, Moon AM, Arenkiel BR, Capecchi MR (2004). The roles of Fgf4 and Fgf8 in limb bud initiation and outgrowth. Dev Biol.

[17] Hernández-Martínez R, Castro-Obregón S, Covarrubias L (2009). Progressive interdigital cell death: regulation by the antagonistic interaction between fibroblast growth factor 8 and retinoic acid. Development.

[18] Al-Qattan MM, Alkuraya FS (2019). Cenani-Lenz syndrome and other related syndactyly disorders due to variants in LRP4, GREM1/FMN1, and APC: insight into the pathogenesis and the relationship to polyposis through the WNT and BMP antagonistic pathways. Am J Med Genet Part A.

[19] Murgai A, Altmeyer S, Wiegand S, Tylzanowski P, Stricker S (2018). Cooperation of BMP and IHH signaling in interdigital cell fate determination. PLoS ONE.

[20] Gros J, Hu JKH, Vinegoni C, Feruglio PF, Weissleder R, Tabin CJ (2010). WNT5A/JNK and FGF/MAPK pathways regulate the cellular events shaping the vertebrate limb bud. Curr Biol.

[21] Montero JA, Hurle JM, Lorda-diez CI, Sanchez-fernandez C. Cell death in the developing vertebrate limb: a locally regulated mechanism contributing to musculoskeletal tissue morphogenesis and differentiation. 2020;1–12. 10.1002/dvdy.237 PMC8451844 32798262

[22] Zhu X-J, Fang Y, Xiong Y, Wang M, Yang X, Li Y (2018). Disruption of Wnt production in *Shh* lineage causes bone malformation in mice, mimicking human Malik–Percin-type syndactyly. FEBS Lett.

[23] Gao B, Song H, Bishop K, Elliot G, Garrett L, English MA (2011). Wnt signaling gradients establish planar cell polarity by inducing vangl2 phosphorylation through Ror2. Dev Cell.

[24] Tian J, Shao J, Liu C, Hou HY, Chou CW, Shboul M (2019). Deficiency of lrp4 in zebrafish and human LRP4 mutation induce aberrant activation of Jagged–Notch signaling in fin and limb development. Cell Mol Life Sci.

[25] Mehawej C, Chouery E, Maalouf D, Baujat G, Le Merrer M, Cormier-Daire V (2012). Identification of a novel causative mutation in the ROR2 gene in a Lebanese family with a mild form of recessive Robinow syndrome. Eur J Med Genet.

[26] Leupin O, Piters E, Halleux C, Hu S, Kramer I, Morvan F (2011). Bone overgrowth-associated mutations in the LRP4 gene impair sclerostin facilitator function. J Biol Chem.

[27] Boudin E, Yorgan T, Fijalkowski I, Sonntag S, Steenackers E, Hendrickx G (2017). The *Lrp4* R1170Q homozygous knock-in mouse recapitulates the bone phenotype of Sclerosteosis in humans. J Bone Miner Res.

[28] Alrayes N, Aziz A, Ullah F, Ishfaq M, Jelani M, Wali A (2020). Novel missense alteration in LRP4 gene underlies Cenani–Lenz syndactyly syndrome in a consanguineous family. J Gene Med.

[29] Sebastian A, Loots GG (2018). Genetics of Sost/SOST in sclerosteosis and van Buchem disease animal models. Metabolism.

[30] Démurger F, Ichkou A, Mougou-Zerelli S, Le Merrer M, Goudefroye G, Delezoide AL (2015). New insights into genotype-phenotype correlation for GLI3 mutations. Eur J Hum Genet.

[31] Shi L, Huang H, Jiang Q, Huang R, Fu W, Mao L (2020). Sub-exome target sequencing in a family with syndactyly type IV due to a novel partial duplication of the LMBR1 gene: first case report in Fujian Province of China. Front Genet.

[32] Xu J, Wu J, Teng X, Cai L, Yuan H, Chen X (2020). Large duplication in LMBR1 gene in a large Chinese pedigree with triphalangeal thumb polysyndactyly syndrome. Am J Med Genet Part A.

[33] Koide T, Hayata T, Cho KWY (2006). Negative regulation of Hedgehog signaling by the cholesterogenic enzyme 7-dehydrocholesterol reductase. Development.

[34] Azoury SC, Reddy S, Shukla V, Deng C-X (2017). Fibroblast growth factor receptor 2 (FGFR2) mutation related syndromic craniosynostosis. Int J Biol Sci.

[35] O’Rourke MP, Soo K, Behringer RR, Hui CC, Tam PPL (2002). Twist plays an essential role in FGF and SHH signal transduction during mouse limb development. Dev Biol.

[36] Zuniga A, Quillet R, Perrin-Schmitt F, Zeller R (2002). Mouse Twist is required for fibroblast growth factor-mediated epithelial-mesenchymal signalling and cell survival during limb morphogenesis. Mech Dev.

[37] Zhao X, Sun M, Zhao J, Leyva JA, Zhu H, Yang W (2007). Mutations in HOXD13 underlie syndactyly type V and a novel brachydactyly-syndactyly syndrome. Am J Hum Genet.

[38] Avitan-Hersh E, Indelman M, Bergman R, Sprecher E (2010). ADULT syndrome caused by a mutation previously associated with EEC syndrome. Pediatr Dermatol.

[39] Alessandri J-L, Dagoneau N, Laville J-M, Baruteau J, Hébert J-C, Cormier-Daire V (2010). *RAB23* mutation in a large family from Comoros Islands with Carpenter syndrome. Am J Med Genet Part A.

[40] Schwarzer W, Witte F, Rajab A, Mundlos S, Stricker S (2009). A gradient of ROR2 protein stability and membrane localization confers brachydactyly type B or Robinow syndrome phenotypes. Hum Mol Genet.

[41] van Lierop AH, Appelman-Dijkstra NM, Papapoulos SE (2017). Sclerostin deficiency in humans. Bone.

[42] Boudin E, Yorgan ÃT, Fijalkowski ÃI, Sonntag S, Steenackers E, Hendrickx G (2017). The Lrp4 R1170Q homozygous knock-in mouse humans. J Bone Miner Res.

[43] Sethi SK, Goyal D, Khalil S, Yadav DK (2013). Two Indian families with Greig cephalopolysyndactyly with non-syndromic phenotype. Eur J Pediatr.

[44] Yousaf M, Azeem Z, Bilal M, Liaqat K, Hussain S (2019). Variants in GLI3 cause Greig Cephalopolysyndactyly syndrome. Genet Test Mol Biomark.

[45] Al-Qattan MM, Shamseldin HE, Al Mazyad M, Al Deghaither S, Alkuraya FS (2013). Genetic heterogeneity in type III familial cutaneous syndactyly and linkage to chromosome 7q36. Am J Med Genet Part A.

[46] Ko JS, Choi BS, Seo JK, Shin JY, Chae JH, Kang GH (2010). A novel DHCR7 mutation in a Smith–Lemli–Opitz syndrome infant presenting with neonatal cholestasis. J Korean Med Sci.

[47] Twigg SRF, Lloyd D, Jenkins D, Elçioglu NE, Cooper CDO, Al-Sannaa N (2012). Mutations in multidomain protein MEGF8 identify a carpenter syndrome subtype associated with defective lateralization. Am J Hum Genet.

[48] Haye D, Collet C, Sembely-Taveau C, Haddad G, Denis C, Soulé N (2014). Prenatal findings in carpenter syndrome and a novel mutation in RAB23. Am J Med Genet Part A.

[49] Jenkins D, Seelow D, Jehee FS, Perlyn CA, Alonso LG, Bueno DF (2007). RAB23 mutations in carpenter syndrome imply an unexpected role for Hedgehog signaling in cranial-suture development and obesity. Am J Hum Genet.

[50] Singh CB, Mishra B, Patel R, Kumar A, Ali A (2021). Tripod-shaped syndactyly in Apert syndrome with FGFR2 p.P253R mutation. Indian J Plast Surg..

[51] Suzuki H, Suda N, Shiga M, Kobayashi Y, Nakamura M, Iseki S (2012). Apert syndrome mutant FGFR2 and its soluble form reciprocally alter osteogenesis of primary Calvarial osteoblasts. J Cell Physiol.

[52] Hackett A, Rowe L (2006). FGFR1 Pfeiffer syndrome without craniosynostosis: an additional case report. Clin Dysmorphol.

[53] Chun K, Teebi AS, Jung JH, Kennedy S, Laframboise R, Meschino WS (2002). Genetic analysis of patients with the Saethre–Chotzen phenotype. Am J Med Genet.

[54] Tian J, Shao J, Liu C, Hou HY, Chou CW, Shboul M (2019). Deficiency of lrp4 in zebrafish and human LRP4 mutation induce aberrant activation of Jagged–Notch signaling in fin and limb development. Cell Mol Life Sci.

[55] Lettice LA, Daniels S, Sweeney E, Venkataraman S, Devenney PS, Gautier P (2011). Enhancer-adoption as a mechanism of human developmental disease. Hum Mutat.

[56] Charzewska A, Obersztyn E, Hoffman-Zacharska D, Lenart J, Poznański J, Bal J (2015). Novel mutations in the IRF6 gene on the background of known polymorphisms in Polish patients with orofacial clefting. Cleft Palate-Craniofacial J.

[57] Rath M, Spiegler S, Strom TM, Trenkler J, Kroisel PM, Felbor U (2019). Identification of pathogenic *YY1AP1* splice variants in siblings with Grange syndrome by whole exome sequencing. Am J Med Genet Part A.

[58] Yigit G, Wieczorek D, Bögershausen N, Beleggia F, Möller-Hartmann C, Altmüller J (2016). A syndrome of microcephaly, short stature, polysyndactyly, and dental anomalies caused by a homozygous *KATNB1* mutation. Am J Med Genet Part A.

[59] Torres L, Hernández G, Barrera A, Ospina S, Prada R (2015). Molecular analysis of exons 8, 9 and 10 of the fibroblast growth factor receptor 2 (FGFR2) gene in two families with index cases of apert syndrome. Colomb Med.

[60] Waye JS, Nakamura LM, Eng B, Hunnisett L, Chitayat D, Costa T (2002). Smith-Lemli-Opitz syndrome: carrier frequency and spectrum of DHCR7 mutations in Canada. J Med Genet.

[61] Potuijt JWP, Baas M, Sukenik-Halevy R, Douben H, Nguyen P, Venter DJ (2018). A point mutation in the pre-ZRS disrupts sonic hedgehog expression in the limb bud and results in triphalangeal thumb–polysyndactyly syndrome. Genet Med.

[62] Díaz-González F, Parrón-Pajares M, Barcia-Ramirez A, Heath KE (2020). First case of compound heterozygous BHLHA9 variants in mesoaxial synostotic syndactyly with phalangeal reduction. Am J Med Genet Part A.

[63] Malik S (2018). Mesoaxial synostotic syndactyly with phalangeal reduction (MSSD): syndactyly type IX. Skelet Radiol.

[64] Malik S, Schott J, Ali SW, Oeffner F, Amin-Ud-Din M, Ahmad W (2005). Evidence for clinical and genetic heterogeneity of syndactyly type I: the phenotype of second and third toe syndactyly maps to chromosome 3p21.31. Eur J Hum Genet.

[65] Malik S, Abbasi AA, Ansar M, Ahmad W, Koch MC, Grzeschik KH (2006). Genetic heterogeneity of synpolydactyly: a novel locus SPD3 maps to chromosome 14q11.2–q12. Clin Genet.

[66] Weinrich JM, Ajabnoor W, Bannas P (2017). Case report of a novel nonsyndromic unilateral syndactyly of the hand. Skeletal Radiol.

[67] Özdemir M, Kavak P, Kaplanoğlu H (2020). Case report an unusual association of ulnar Hemimelia with mesoaxial synostotic syndactyly. BJR Case Rep..

[68] Bosse K, Betz RC, Lee YA, Wienker TF, Reis A, Kleen H (2000). Localization of a gene for syndactyly type 1 to chromosome 2q34-q36. Am J Hum Genet.

[69] Ghadami M, Majidzadeh-A K, Haerian BS, Damavandi E, Yamada K, Pasallar P (2001). Confirmation of genetic homogeneity of syndactyly type 1 in an Iranian family. Am J Med Genet.

[70] Montagu MFA (1953). A pedigree of Syndactylism of the middle and ring fingers. Hum Genet..

[71] Castilla EE, Paz JE, Orioli-Parreiras IM (1980). Syndactyly: Frequency of specific types. Am J Med Genet.

[72] Muragaki Y, Mundlos S, Upton J, Olsen BR (1996). Altered growth and branching patterns in synpolydactyly caused by mutations in HOXD13. Science (80-)..

[73] Debeer P, Schoenmakers EFPM, Twal WO, Argraves WS, De Smet L, Fryns JP (2002). The fibulin-1 gene (FBLN1)is disrupted in a t(12;22) associated with a complex type of synpolydactyly. J Med Genet.

[74] Ngoc NT, Duong NT, Quynh DH, Ton ND, Duc HH, Huong LTM (2020). Identification of novel missense mutations associated with non-syndromic syndactyly in two vietnamese trios by whole exome sequencing. Clin Chim Acta.

[75] Dobrowolski R, Sasse P, Schrickel JW, Watkins M, Kim JS, Rackauskas M (2008). The conditional connexin43G138R mouse mutant represents a new model of hereditary oculodentodigital dysplasia in humans. Hum Mol Genet.

[76] Johnston O, Kirby VV (1955). Syndactyly of the ring and little finger. Am J Hum Genet..

[77] Haas SL (1940). Bilateral complete syndactylism of all fingers. Am J Surg.

[78] Wieczorek D, Pawlik B, Li Y, Akarsu NA, Caliebe A, May KJW (2010). A specific mutation in the distant sonic hedgehog (SHH) cis-regulator (ZRS) causes Werner Mesomelic Syndrome (WMS) while complete ZRS duplications underlie Haas type polysyndactyly and preaxial polydactyly (PPD) with or without triphalangeal thumb. Hum Mutat.

[79] Wu L, Liang D, Niikawa N, Ma F, Sun M, Pan Q (2009). A ZRS duplication causes Syndactyly type IV with Tibial Hypoplasia. Am J Med Genet Part A.

[80] Cn D (1896). Cleft hand: a report of a case successfully treated by the use of periosteal flaps. Ann Surg.

[81] Temtamy SA, McKusick VA. The genetics of hand malformations. 1978. 215242

[82] Li Y, Pawlik B, Elcioglu N, Aglan M, Kayserili H, Yigit G (2010). LRP4 mutations Alter Wnt/β-catenin signaling and cause limb and kidney malformations in Cenani–Lenz syndrome. Am J Hum Genet.

[83] Dimitrov BI, Voet T, De Smet L, Vermeesch JR, Devriendt K, Fryns JP (2010). Genomic rearrangements of the GREM1-FMN1 locus cause oligosyndactyly, radio-ulnar synostosis, hearing loss, renal defects syndrome and Cenanie–Lenz-like non-syndromic oligosyndactyly. J Med Genet.

[84] Jamsheer A, Zemojtel T, Kolanczyk M, Stricker S, Hecht J, Krawitz P (2013). Whole exome sequencing identifies FGF16 nonsense mutations as the cause of X-linked recessive metacarpal 4/5 fusion. J Med Genet.

[85] Jones B, Byers H, Stewart Watson J, Newman WG (2014). Identification of a novel familial FGF16 mutation in metacarpal 4–5 fusion. Clin Dysmorphol.

[86] Kantaputra PN, Carlson BM (2019). Genetic regulatory pathways of split-hand/foot malformation. Clin Genet.

[87] Khan A, Wang R, Han S, Ahmad W, Zhang X (2017). A novel homozygous missense mutation in BHLHA9 causes mesoaxial synostotic syndactyly with phalangeal reduction in a Pakistani family. Hum Genome Var.

[88] Adkinson JM, Chung KC. Release of Finger Syndactyly Using Dorsal Rectangular Flap. Oper Tech Hand Wrist Surg. 2018. p. 811–8.

[89] Boczek NJ, Miller EM, Ye D, Nesterenko VV, Tester DJ, Antzelevitch C (2015). Novel Timothy syndrome mutation leading to increase in CACNA1C window current. Hear Rhythm.

[90] Kim H-G, Rosenfeld JA, Scott DA, Bénédicte G, Labonne JD, Brown J (2019). Disruption of PHF21A causes syndromic intellectual disability with craniofacial anomalies, epilepsy, hypotonia, and neurobehavioral problems including autism. Mol Autism..

[91] Orge FH, Dar SA, Blackburn CN, Grimes-Hodges SJ, Mitchell AL (2016). Ocular manifestations of X-linked dominant FAM58A mutation in toe syndactyly, telecanthus, anogenital, and renal malformations (‘STAR’) syndrome. Ophthalmic Genet.

[92] Vladimir Socolov R, Ioana Andreescu N, Maria Haliciu A, Vlad Gorduza E, Dumitrache F, Anca Balan R (2015). Intrapartum diagnostic of Roberts syndrome-case presentation. Rom J Morphol Embryol.

[93] Struck TJ, Mannakee BK, Gutenkunst RN (2018). The impact of genome-wide association studies on biomedical research publications. Hum Genom.

